# A mutation responsible for impaired detection by the Xpert SARS-CoV-2 assay independently emerged in different lineages during the SARS-CoV-2 pandemic

**DOI:** 10.1186/s12866-023-02924-8

**Published:** 2023-07-17

**Authors:** Daniel Peñas-Utrilla, Amadeo Sanz, Pilar Catalán, Cristina Veintimilla, Luis Alcalá, Roberto Alonso, Patricia Muñoz, Laura Pérez-Lago, Darío García de Viedma, on behalf of the Gregorio Marañón Microbiology-ID COVID 19 Study Group, Teresa Aldámiz, Ana Álvarez-Uría, Elena Bermúdez, Emilio Bouza, Sergio Buenestado-Serrano, Almudena Burillo, Raquel Carrillo, Pilar Catalán, Emilia Cercenado, Alejandro Cobos, Cristina Díez, Pilar Escribano, Agustín Estévez, Chiara Fanciulli, Alicia Galar, Mª Dolores García, Paloma Gijón, Helmuth Guillén, Jesús Guinea, Marta Herranz, Álvaro Irigoyen, Martha Kestler, Juan Carlos López, Marina Machado, Mercedes Marín, Pablo Martín-Rabadán, Andrea Molero-Salinas, Pedro Montilla, Belén Padilla, Rosalía Palomino-Cabrera, María Palomo, María Jesús Pérez-Granda, Leire Pérez, Elena Reigadas, Cristina Rincón, Belén Rodríguez, Sara Rodríguez, Cristina Rodríguez-Grande, Adriana Rojas, María Jesús Ruiz-Serrano, Carlos Sánchez, Mar Sánchez, Julia Serrano, Francisco Tejerina, Maricela Valerio, Mª Cristina Veintimilla, Lara Vesperinas, Teresa Vicente, Sofía de la Villa

**Affiliations:** 1 Servicio de Microbiología Clínica y Enfermedades Infecciosas, Hospital General Universitario Gregorio Marañón, C/Dr. Esquerdo 46, 28007 Madrid, Spain; 2 Instituto de Investigación Sanitaria Gregorio Marañón (IiSGM), Madrid, Spain; 3 Departamento de Medicina, Universidad Complutense, Madrid, Spain; 4 Centro de Investigación Biomédica en Red (CIBER) de Enfermedades Respiratorias (CIBERES), Madrid, Spain; 5 Centro de Investigación Biomédica en Red (CIBER) de Enfermedades Infecciosas (CIBERINFEC), Madrid, Spain

**Keywords:** COVID-19, Cepheid Xpert, C29200T, Detection impairment, N gene

## Abstract

**Background:**

COVID-19 diagnosis lies on the detection of SARS-CoV-2 on nasopharyngeal specimens by RT-PCR. The Xpert-Xpress SARS-CoV-2 assay provides results in less than one hour from specimen reception, which makes it suitable for clinical/epidemiological circumstances that require faster responses. The analysis of a COVID-19 outbreak suspected in the neonatology ward from our institution showed that the Ct values obtained for the targeted genes in the Xpert assay were markedly different within each specimen (N Ct value > 20 cycles above the E Ct value).

**Results:**

We identified the mutation C29200T in the N gene as responsible for an impairment in the N gene amplification by performing whole genome sequencing of the specimens involved in the outbreak (Omicron variant). Subsequently, a retrospective analysis of all specimens sequenced in our institution allowed us to identify the same SNP as responsible for similar impairments in another 12 cases (42% of the total cases reported in the literature). Finally, we found that the same SNP emerged in five different lineages independently, throughout almost all the COVID-19 pandemic.

**Conclusions:**

We demonstrated for the first time the impact of this SNP on the Xpert assay, when harbored by new Omicron variants. We extend our observation period throughout almost all the COVID-19 pandemic, offering the most updated observations of this phenomenon, including sequences from the seventh pandemic wave, until now absent in the reports related to this issue. Continuous monitoring of emerging SNPs that could affect the performance of the most commonly used diagnostic tests, is required to redesign the tests to restore their correct performance.

## Background

The diagnosis of COVID-19 is based on RT-PCR detection of SARS-CoV-2 in nasopharyngeal (NP) specimens. Many different PCR designs have been developed, simultaneously targeting different viral genomic targets, such as TaqPath COVID-19 CE-IVD RT-PCR kit (Thermo Fisher Scientific) or Xpert Xpress SARS-CoV-2 assay (Cepheid, CA). Due to laboratory workload during the pandemic, it has taken between several hours and a day before RT-PCR results become available, including time spent on nucleic acid extraction and purification. At our institution, in clinical/epidemiological scenarios where more rapid results are needed, the Xpert Xpress SARS-CoV-2 assay (Cepheid, CA) is applied, since it offers a RT-PCR result within 30–45 min from receipt of the sample. The test combines extraction and RT-PCR in the same cartridge and targets two viral genes (the envelope (E) and nucleocapsid (N2) genes), together with an internal control. In some cases, the Ct values obtained for the targeted genes with the Xpert Xpress SARS-CoV-2 assay are markedly different. In this work, we aim to study the cause of this impairment detection and try to contextualise it during the COVID-19 pandemic.

## Results and Discussion

In June 2022, an outbreak of COVID-19 was suspected in the neonatal ward at our institution, involving three neonates and two healthcare workers (HCWs). For rapid characterization of this alert, Xpert tests were performed on NP specimens from the three neonates. A non-conventional result was obtained in all three cases, with a marked difference in cycle threshold (Ct) values obtained for the E and N genes in the same specimen (N Ct value > 20 cycles above the E Ct value; Table 1, specimens 1–3). When a second specimen from all three neonates was retested by Xpert, the same results were reproduced, whereas an analysis of the specimens applying the routine test used in our laboratory (TaqPath, Thermofisher, MAS, USA) did not detect significant differences between the Ct values of the target genes (N, S and ORF1ab). Our interpretation was that a mutation in the N gene may had been preventing the correct hybridization of the probe targeting the N gene in the Xpert test, and that we were probably facing an outbreak strain, since the specimens from the three neonates tested shared the same abnormal result by Xpert assay.

**Table 1:** SARS-CoV-2 specimens harboring the C29200T SNP at our institution

ENA accession no	Specimen no	Date of collection	Lineage	N Gene Ct (Xpert)	E Gene Ct (Xpert)
ERS14365539	1	2022/06/21	BA.5.1	> 45	18.4
ERS14365546	2	2022/06/24	BA.5.1	44.8	23.2
ERS14365547	3	2022/06/24	BA.5.1	> 45	19.6
ERS14365540	4	2020/12/08	B.1.1.141	> 45	21.2
ERS14365541	5	2021/07/21	B.1.621	> 45	16.1
ERS14365542	6	2021/06/19	B.1.621	> 45	15.8
ERS14365543	7	2021/06/23	B.1.621	> 45	12.3
ERS14365544	8	2021/07/01	B.1.621	40.8	19.5
ERS14365549	9	2021/09/20	AY.122	> 45	15.1
ERS14365545	10	2022/01/29	BA.1.1	43.4	21.5
ERS14365538	11	2022/02/15	BA.2	> 45	27.3
ERS14365549	12	2022/07/07	BA.5.1	40.3	17.4

To evaluate our hypothesis, we performed whole genome sequencing (WGS) [1] of the specimens taken from the three neonates and two HCWs suspected to be involved in the outbreak. The specimens from the neonates were identified in all three cases as being the same Omicron BA.5.1 strain (0 SNPs between them), confirming patient-to-patient transmission (outbreak strain). The involvement of the two HCWs (who had not been tested by Xpert) was ruled out because they showed a 4 and 6 SNP difference with respect to the outbreak strain, which left the question of how the outbreak strain had entered the ward unresolved. A more detailed analysis of SNPs called in the N gene in the outbreak strain indicated that, after excluding the six BA.5.1 marker SNVs [C28311T (P13L), -28,362- (DEL31/33), G28881A (R203K), G28882A (R203K), G28883C (G204R) and A29510C (S413R)] (https://outbreak.info/), the outbreak strain harbored only one other SNV in the N gene (the synonymous mutation C29200T). This SNP was, therefore, the candidate responsible for impaired detection of the N gene by Xpert assay.

We then explored whether this SNP could also be found during the pandemic as an independent and emergent mutation in lineages preceding the BA.5.1 reported here. For this, we used the MAFFT program to perform multiple sequence alignment [2, 3] of all 9364 sequences obtained in our population during all pandemic waves (March 2020 to August 2022). This led to the identification of 9 specimens (specimens 4 –12; Table 1) carrying this same SNP.

**Fig. 1:**
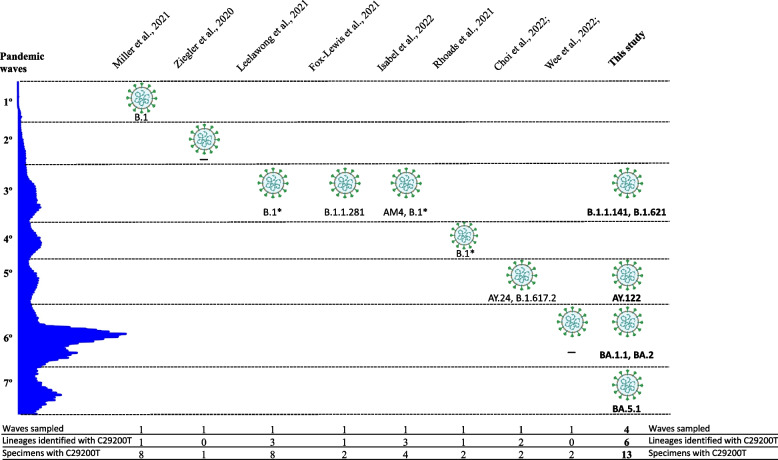
Graphic representation of the findings (waves sampled, number of lineages and specimens identified harboring it) extracted from the literature and from our study regarding C29200T SNP

These corresponded to six different lineages, B.1.1.141, B.1.621, AY.122, BA.1.1, BA.2 and BA.5.1, from specimens taken in the third, fifth, sixth and seventh pandemic waves, respectively (Table 1 and Fig. 1). These findings, taken together, indicated that we were facing an independent and emergent event that occurred independently in different lineages and at different times during the various pandemic waves. A review of the results returned when these 9 specimens were tested indicated that 5 of them had been assayed by Xpert; a marked deviation between Ct values for the N and E genes had been found and they had been reported as positive. Ct values for the target genes in the 4 remaining specimens, which had been tested by TaqPath, were consistent with each other but, when retested now by Xpert, impaired detection in the N gene was observed (Table 1, specimens 4, 6, 8 and 11).

Finally, since the impact of the C29200T SNP on the N gene Ct value was not always the same for the strains harboring it (Table 1), we assessed whether some specimens with less marked differences between Xpert Ct values for the N and E genes, might have been overlooked. Given that the difference in Ct values between N and E genes in our sample was 21–33, we narrowed the range (difference between N and E Ct values < 20) to retrospectively capture additional potential candidates for SNP inclusion. We found 33 specimens that met this criterion (Ct differences ranged between 6 and 12) and we sequenced the six with the greatest Ct difference values of those available. The C29200T SNP was not detected in any of them. This indicates that a Ct difference > 20 between N and E values constitutes a specific feature predictive of the presence of the C29200T SNP, whereas lower Ct differences seem to correspond to intrinsic intra-assay aberrations, not to the presence of an impairing mutation.

Our findings mean that impaired Xpert performance due to C29200T affected 0.13% (12/9364) of the total sequences obtained from the population covered by our hospital. A similar percentage (0.15%) was found when we calculated the proportion of the C29200T mutation in Spain, after analyzing all Spanish SARS-CoV-2 genomes deposited in GISAID submitted between 12 March 2020 and 25 August 2022 (225 out of 150,441 sequences).

A review of the literature (Fig. 1) enabled us to determine that the same SNP C29200T [4–11], as well as three different SNPs (G29140T [12] C29200A [13], C29197T [5–8, 14–16], C29203T [10]) in the N gene, had been reported as responsible for alterations in Xpert detection. The first description of SNP C29200T was in July 2020 [4], in Germany (involving a single specimen). Since that first description, no other findings from Europe have been reported and the remaining descriptions (28 cases in total) are restricted to the USA [5, 6, 10], Canada [9], New Zealand [7], Singapore [11] and Australia [8]. It was shown that nearly all the cases reported by impaired Xpert assay due to the C29200T SNP were restricted to the B1 lineage, with the exception of four Delta sequences. The reported data covered the first through sixth pandemic waves, with the last sequence reported in January 2022. However, no data were found either for the last pandemic wave or the Omicron lineage (Fig. 1).

Apart from the anecdotal description of the first sequence in Germany [4], our data (Fig. 1) represent the only study in Europe focused on this SNP and its impact on diagnosis. We add valuable data to the scant details devoted to the characterization of this event in the literature. We provide 12 new observations of the C29200T mutation (42% of the total sequences reported in the literature); we also present data of the SNP emerging independently in five different lineages, and demonstrate for the first time its presence and impact on the Xpert assay for the new Omicron variants (Fig. 1). Our observation period extends across almost the entire COVID-19 pandemic, offering the most up-to-date observations of this phenomenon, including sequences from the seventh pandemic wave, hitherto absent in reports on this issue. Finally, despite the low frequency of this SNP, a strain harboring it was involved in an outbreak at our institution, and the impaired detection by Xpert common to the cases was a feature (preceding WGS data) that supported the suspicion of outbreak. The same N-gene dropout in Xpert was also recently used as a diagnostic feature to suspect a nosocomial outbreak in Singapore [11].

Our data indicate that the C29200T SNP is not linked to a specific lineage that emerges individually, but has independently emerged in many different lineages during the pandemic, which perpetuates the possibility of impaired detection when applying RT-PCR tests. Here we focused on the impact on the Xpert assay, although similar phenomena could affect any other assay, depending on the regions targeted and the probes designed to interrogate them. In May 2022, the company marketing the GeneXpert family of systems released a new kit (Xpert Xpress CoV-2 plus) that corrects the failure to detect the N gene caused by the C29200T variant described here. Our study exemplifies the usefulness of continually monitoring the emergence of SNPs that could affect the performance of the most commonly used diagnostic tests, alerting and guiding manufacturers to redesign the tests to restore their correct performance.

## Methods

### SARS-CoV-2 RT-PCR based detection

The material for analysis corresponded to the stored remnants of nasopharyngeal swabs that had been taken for diagnostic purposes. RNA was purified from 300 μL of nasopharyngeal exudate in a KingFisher (Thermo Fisher Scientific) equipment. Cepheid Xpert Xpress SARS-CoV-2 Assay (Cepheid, CA) and TaqPath COVID-19 CE-IVD RT-PCR kit (Thermo Fisher Scientific) were applied for SARS-CoV-2 RT-PCR based detection as per the manufacturer’s instructions.

### Illumina sequencing

Sixteen μL of RNA was used for reverse transcription by LunaScript RT SuperMix Kit (New England BioLabs). Whole-genome amplification of the coronavirus was performed with the Artic nCoV-2019 V3 and V4 panel of primers (Integrated DNA Technologies, artic.network/ncov-2019) and Q5 Hot Start DNA polymerase (New England BioLabs). Libraries were prepared using the DNA Prep Kit (Illumina), following the manufacturer’s instructions. Libraries were quantified with the Quantus Fluorometer (Promega) and then pooled at equimolar concentrations (4 nM). Finally, pooled libraries were sequenced on a MiSeq instrument (V2 flow cell).

An in-house bioinformatic pipeline was applied to analyse sequencing data (https://github.com/MG-IiSGM/covid_multianalysis). Adapters and low-quality regions were trimmed from paired reads using fastp (version 0.20.1), a tool for ultra-fast all-in-one preprocessing for raw FASTQ files, considering a mean quality of 20. Quality control was then assessed with fastQC (version v0.11.9), a tool that provides a modular set of analyses to quickly and easily assess the quality of sequencing data, with default parameters. Good quality reads were mapped with BWA (version 0.7.17-r1188), a software package for mapping low-divergent sequences against a large reference genome, using default parameters, to the Wuhan-1 SARS-CoV-2 sequence (GenBank accession no. NC_045512.2) as a reference. Duplicate reads in the alignment files were then removed using picard (2.27.4-SNAPSHOT), a set of command line tools for manipulating high-throughput sequencing data. Variant calling and consensus sequence generation was performed with IVAR (version 1.3.1), a computational package that contains functions broadly useful for viral amplicon-based sequencing. SNPs with a mean depth of 15X and a frequency higher than 70% were considered. Lineage annotation was performed with pangolin (version v4.1.2), a software package for assigning SARS-CoV-2 genome sequences to global lineages. MAFFT (version v7.471) was used to perform multiple sequence analysis.

## Data Availability

The data (fastq files) that support the findings of this study are available at ENA (https://www.ebi.ac.uk) under the project accession number PRJEB58553; the accession number of the sequenced strains used in the study can be found in Table 1.
